# Regulating Effect of Mixed Cropping of Green Manure Varieties on Greenhouse Gas Emissions in a Green Manure–Wheat Rotation System Under Reduced Nitrogen Conditions

**DOI:** 10.3390/plants15142194

**Published:** 2026-07-17

**Authors:** Xingjian Jin, Ke Xu, Zhengpeng Li, Xiaojun Wang, Qingbiao Yan, Kaibin Qi, Falong Hu, Tianlong Chen, Mei Han

**Affiliations:** 1 College of Agriculture and Animal Husbandry, Qinghai University, Xining 810016, China; a1941002731@163.com (X.J.);; 2 Academy of Agriculture and Forestry Sciences, Qinghai University, Xining 810016, China; 3 College of Agronomy, Gansu Agricultural University, Lanzhou 730070, China; 4 Academy of Animal Science and Veterinary, Qinghai University, Xining 810016, China

**Keywords:** Qinghai Plateau, nitrogen reduction, mixed sown green manure, spring wheat, greenhouse gas emissions, soil physicochemical properties

## Abstract

To explore low-carbon spring wheat production strategies for the ecologically fragile Qinghai Plateau, a two-year field experiment was conducted using a split-plot design. The main plots were set with three nitrogen (N) application levels: N0 (0 kg·ha^−1^, no N input), N1 (157.5 kg·ha^−1^, 30% N reduction relative to conventional rate), and N2 (225 kg·ha^−1^, the conventional rate). The subplots comprised four green manure mixed cropping patterns: sole cropped common vetch (CK), common vetch mixed with hull-less barley (HB), common vetch mixed with hairy vetch (HV), and common vetch mixed with rapeseed (RS). Averaged across green manure treatments, N1 reduced global warming potential (GWP) by 14.56% and greenhouse gas intensity (GHGI) by 16.89% compared with N2. Under the N1 treatment, compared with CK, RS reduced cumulative CO_2_ emissions by 7.77% and GWP by 7.45%, maintained wheat grain yield comparable to those under N2, and obtained the minimum GHGI which was 2.92% lower than CK. Regarding soil properties, all green manure patterns significantly increased soil available phosphorus (AP) and available potassium (AK) compared with CK. Specifically, HB increased AP by 12.81% and AK by 16.38%, and RS also increased AP by 4.37% and AK by 2.36%. In addition, HB enhanced soil organic matter (SOM) by 5.85%, ammonium nitrogen (NH_4_–N) by 8.13%, and nitrate nitrogen (NO_3_–N) by 4.34%. Soil NH_4_–N and NO_3_–N were identified as key drivers of GHGI across treatments. On the Qinghai–Tibet Plateau, a 30% N reduction combined with target-oriented green manure mixtures—RS for emission mitigation and HB for high yield and fertility—offers a viable low-carbon strategy for spring wheat production.

## 1. Introduction

Agricultural ecosystems are major sources of greenhouse gas (GHG) emissions. Annual emissions of CO_2_, CH_4_, and N_2_O from global croplands account for approximately 10–12% of total anthropogenic GHG emissions [1,2]. Among these gases, N_2_O has a global warming potential (GWP) approximately 298 times that of CO_2_ and is primarily generated through soil nitrification and denitrification processes [3,4]. In contrast, CH_4_, with a GWP 27 times that of CO_2_, is mainly absorbed by upland soils via methane-oxidizing bacteria [3,5]. As global food demand continues to rise, excessive nitrogen (N) fertilizer application has become a critical driver of agricultural GHG emissions [6,7]. Numerous studies have shown that surplus N inputs not only stimulate N_2_O emissions through nitrification and denitrification but also alter microbial activity by modifying soil C/N ratios, thereby affecting CO_2_ efflux and CH_4_ oxidation capacity [8,9]. Therefore, identifying feasible strategies to reduce N fertilizer use while ensuring food security is a key scientific challenge for promoting green and low-carbon agricultural transformation [10].

Green manure is an important organic fertilizer source in agricultural ecosystems, with well-documented benefits for soil fertility improvement, mineral fertilizer substitution, and enhancement of soil physicochemical properties [11,12,13,14]. In regions with surplus heat and light resources, planting green manure after wheat harvest offers significant ecological and productive advantages. Studies have shown that green manure incorporation can replace 20–40% of mineral N fertilizer for the subsequent crop, effectively reducing fertilizer input [15,16]. Additionally, green manure returning adds exogenous organic carbon, promotes soil aggregate formation, enhances physical protection of organic carbon, and thereby influences CO_2_ release dynamics [17,18,19,20]. In terms of N transformation, the high C/N ratio of green manure promotes microbial immobilization of inorganic N, reducing substrate availability for nitrification and denitrification, thus lowering N_2_O emissions [21,22]. Furthermore, root exudates of green manure can regulate the community structure of methanotrophs and methanogens in the soil, affecting CH_4_ uptake and emission balance [23,24,25,26].

Mixed cropping common vetch (*Vicia sativa* L.) with gramineous (e.g., hull-less barley) or cruciferous (e.g., rapeseed) species maintains the N-fixing capacity of legumes. The high C/N ratio of gramineous species regulates the decomposition rate of the overall green manure mixture, thereby avoiding rapid N mineralization and subsequent N loss after legume monoculture incorporation [27,28]. Moreover, crop mixtures with different root architectures can improve soil pore structure and aeration, thereby influencing CH_4_ oxidation and N_2_O production [29,30]. Studies have shown that mixed cropping and green manure mixed cropping systems can regulate the abundance and expression of denitrification functional genes (e.g., *nirK*, *nosZ*) in the rhizosphere, thereby influencing N_2_O emissions [31,32]. Similarly, the mixed incorporation of Chinese milk vetch (*Astragalus sinicus* L.) and rapeseed significantly increased soil labile organic carbon and microbial biomass carbon while reducing CH_4_ emission intensity [33]. These findings suggest that optimizing green manure mixtures is an effective approach to enhance GHG mitigation potential.

The Qinghai Plateau, located in the northeastern part of the Qinghai–Tibet Plateau, is characterized by a continental semi-arid climate with low annual temperatures, high evaporation, and slow soil organic matter mineralization. It serves as a crucial ecological buffer and grain production base in China [34]. In this region, wheat production has long suffered from high N fertilizer application levels and low use efficiency, where excess N not only increases production costs but also exacerbates GHG emissions. Meanwhile, after wheat harvest, there remains a surplus of heat and light resources, and the long autumn fallow period is suitable for green manure cultivation [35,36]. In recent years, systematic research on post-wheat green manure planting has been conducted on the Qinghai Plateau, clarifying the adaptability and fertility effects of green manure species such as common vetch, hairy vetch, and hull-less barley [37,38,39]. Existing studies have typically set the conventional N application level for spring wheat at 225 kg·N·ha^−1^ (the local farmers’ practice), with a corresponding reduced-N treatment at 157.5 kg·N·ha^−1^ (i.e., a 30% reduction from the conventional rate) to evaluate the compensatory contribution of green manure to mineral N fertilizer [40,41].

Little is known about how green manure mixed cropping patterns combined with reduced N application affect GHG emissions and wheat yield in spring wheat systems on the Qinghai Plateau, especially the synergistic mechanisms involving soil physicochemical properties. We therefore hypothesize that, under reduced N, common vetch mixed cropping patterns will significantly decrease GHG emissions (particularly N_2_O) while maintaining or increasing yield, compared with conventional full-N monoculture. This synergy is primarily attributed to improved soil properties (e.g., higher C/N ratio, greater aggregate stability, optimized N availability) that reduce N losses and enhance plant N uptake. To test this hypothesis, we established common vetch sole cropping and mixed cropping patterns under three N levels in a spring wheat system and systematically investigated their effects on GHG emissions. The aim is to clarify the potential for a win–win outcome between wheat yield and carbon mitigation, and to elucidate the underlying soil physicochemical mechanisms, thereby providing a theoretical basis for GHG mitigation in regional wheat production.

## 2. Results

### 2.1. Effects of Different N Levels and Green Manure Mixed Cropping Patterns on Wheat Yield

**Figure 1:**
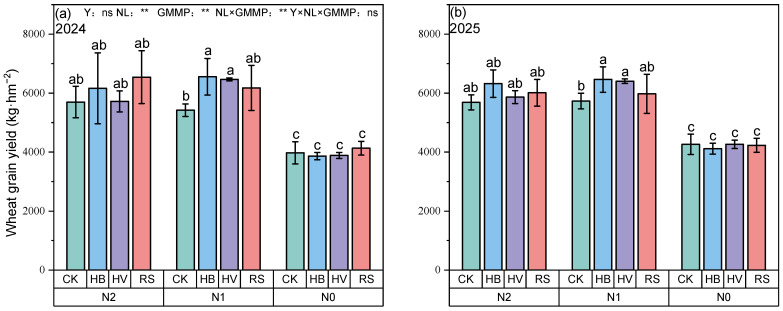
Wheat grain yield under different treatments in 2024 (**a**) and 2025 (**b**). Different lowercase letters denote significant differences at *p* < 0.05 (Tukey’s HSD test, marginal means estimated from linear mixed models). Notes: **, and ns represent *p* < 0.01, and not significant, respectively. Y, year; NL, N application level; GMMP, green manure mixed cropping pattern. N0, N1 and N2 indicate zero N input, 30% N reduction, and conventional N application, respectively. CK, HB, HV and RS represent sole cropped common vetch, common vetch mixed cropped with hull-less barley, common vetch mixed cropped with hairy vetch, and common vetch mixed cropped with rapeseed, respectively.

Linear mixed-effects model (LMM) analysis of the two-year split-plot data revealed that N level had a highly significant main effect on wheat yield (*p* < 0.01), as did green manure pattern (*p* < 0.01), and their two-way interaction was also significant (*p* < 0.01). As shown in Figure 1, neither the experimental year nor the three-way year × N level × green manure interaction was significant (*p* > 0.05), indicating that yield responses to all treatments remained consistent between 2024 and 2025. Across the two experimental years, averaging over all green manure mixed cropping patterns, wheat yield under N1 was 50.26% higher than under N0, with no significant difference between N1 and N2. Averaged over all N levels, relative to sole cropped CK, mixed cropping with HB, HV, and RS increased wheat yield by 8.81%, 5.95%, and 7.46%, respectively. Regarding the two-way interaction, N1HB and N1HV produced higher yields than N2HB and N2HV, although the differences were not statistically significant. Moreover, compared with N1CK, the yields of N1HB and N1HV were 16.72% and 15.41% higher, respectively. Overall, reducing N fertilizer and adopting suitable green manure mixed cropping patterns (particularly HB and HV) offer a promising approach to enhancing wheat yield.

### 2.2. Effects of Different N Levels and Green Manure Mixed Cropping Patterns on Greenhouse Gas Emissions

**Figure 2:**
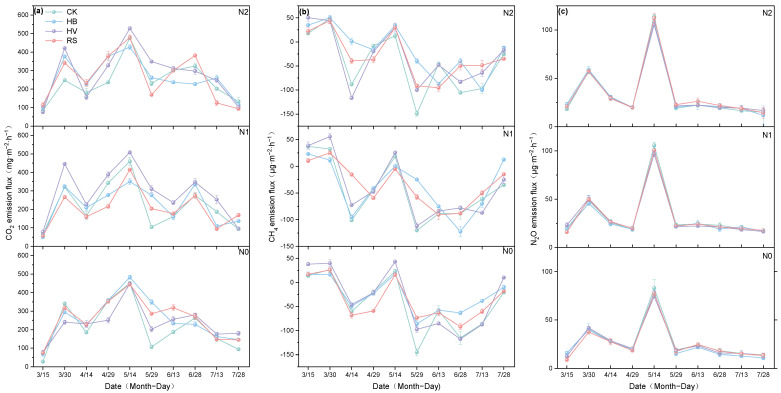

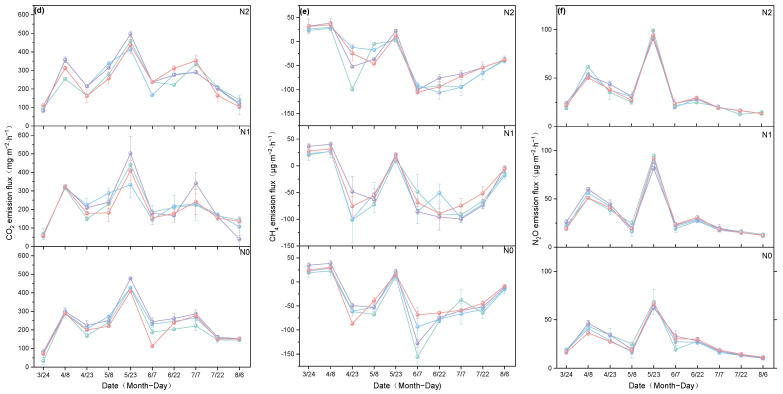
Time-course of soil CO_2_, CH_4_, and N_2_O emission fluxes under different N levels and green manure mixed cropping patterns in 2024 (Panels (**a**–**c**)) and 2025 (Panels (**d**–**f**)). Notes: N0, N1 and N2 indicate zero N input, 30% N reduction, and conventional N application, respectively. CK, HB, HV and RS represent sole cropped common vetch, common vetch mixed cropped with hull-less barley, common vetch mixed cropped with hairy vetch, and common vetch mixed cropped with rapeseed, respectively.

As shown in Figure 2, the soil CO_2_ and N_2_O emission fluxes exhibited an initial increase followed by a decrease during the wheat growing season. The first emission peaks of soil CO_2_ and N_2_O occurred approximately 30 days after wheat sowing, and the second emission peaks appeared after topdressing. In contrast, soil CH_4_ functioned as a sink throughout the wheat growing period, with its CH_4_ uptake flux rising first and then declining. The first CH_4_ uptake peak emerged around 30 days post sowing, and the second uptake peak was observed at roughly 75 days after wheat planting.

Cumulative greenhouse gas emissions are shown in Table 1, for cumulative CO_2_ emissions, LMM analysis showed that the main effects of N application level and green manure mixed cropping pattern, their two-way interaction, and the three-way interaction of year, N application level, and green manure mixed cropping pattern were all highly significant (*p* < 0.01), whereas the main effect of year had no significant effect (*p* > 0.05). Over the two experimental years, across all green manure mixed cropping patterns, compared with N0, the N1 treatment reduced cumulative CO_2_ emissions by 6.06%; compared with N2, N1 reduced cumulative CO_2_ emissions by 14.84%. Averaged across all N levels, compared with CK, the HB, HV, and RS treatments increased cumulative emissions by 6.56%, 10.69%, and 2.38%, respectively. The interaction between N application level and green manure mixed cropping pattern was highly significant, indicating that the responses of green manure patterns to N levels were differed. At N2, cumulative CO_2_ emissions under HB, HV, and RS were 6.61%, 12.31%, and 6.22% higher than under CK, respectively. At N0, the corresponding increases were 13.02%, 11.00%, and 8.36%, respectively. In contrast, at N1, only HV showed a significant increase (8.58% above CK), whereas HB was not significantly different from CK (only 0.11% higher), and RS exhibited a significant reduction of 7.77% compared with CK. Averaged across green manure patterns, the N1 treatment reduced CO_2_ emissions by 14.12% relative to N2 and by 2.91% relative to N0. These findings suggest that a 30% N reduction combined with an appropriate green manure pattern—particularly RS—can effectively mitigate soil CO_2_ emissions while maintaining the agronomic benefits of green manure incorporation.

For cumulative CH_4_ uptake, LMM analysis showed that the main effects of N application level and green manure mixed cropping pattern, their two-way interaction, and the three-way interaction of year, N application level, and green manure mixed cropping pattern were all highly significant (*p* < 0.01), and the main effect of year showed a significant effect (*p* < 0.05). Over the two experimental years, across all green manure mixed cropping patterns, compared with N0, the N1 treatment increased cumulative CH_4_ uptake by 10.25%, while the N2 treatment decreased cumulative CH_4_ uptake by 3.39%; compared with N2, N1 increased cumulative CH_4_ uptake by 14.13%. The sub-treatment (green manure mixed cropping pattern) had a highly significant effect on cumulative CH_4_ uptake (*p* < 0.01). Averaged across all N levels, compared with CK, the HB, HV, and RS treatments decreased cumulative CH_4_ uptake by 19.77%, 16.74%, and 21.89%, respectively. The interaction between N application level and green manure mixed cropping pattern was highly significant, indicating that the response of green manure mixed cropping patterns to different N levels varied. Under the three N levels (N2, N1, and N0), the cumulative CH_4_ uptake of HB, HV, and RS all decreased compared with CK, among which the N1HB treatment showed the smallest reduction and was not significantly different from N1CK. These results suggest that reducing N application by 30% combined with an appropriate green manure mixed cropping pattern can further enhance soil CH_4_ uptake.

For cumulative N_2_O emissions, LMM analysis showed that the main effects of N application level and green manure mixed cropping pattern were highly significant (*p* < 0.01), whereas the main effect of year, their two-way interaction, and the three-way interaction of year, N application level, and green manure mixed cropping pattern had no significant effect (*p* > 0.05). Over the two experimental years, across all green manure mixed cropping patterns, compared with N0, the N1 treatment increased cumulative N_2_O emissions by 19.32%, and the N2 treatment increased cumulative N_2_O emissions by 25.33%; compared with N2, N1 decreased cumulative N_2_O emissions by 4.79%. Averaged across all N levels, compared with CK, the HB, HV, and RS treatments decreased cumulative emissions by 3.83%, 0.94%, and 1.68%. These results suggest that reducing N application by 30% alone is an effective measure to reduce N_2_O emissions, while the choice of green manure mixed cropping pattern has no additional regulatory effect on N_2_O emissions.

### 2.3. Effects of Different N Levels and Green Manure Mixed Cropping Patterns on GWP and GHGI

**Figure 3:**
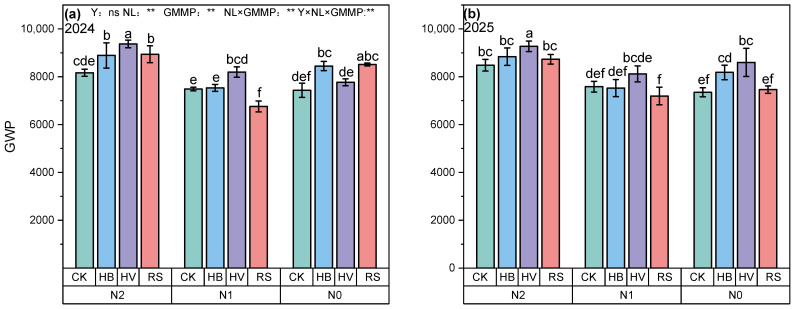
Global warming potential (GWP) under different treatments in 2024 (**a**) and 2025 (**b**). Different lowercase letters denote significant differences at *p* < 0.05 (Tukey’s HSD test, marginal means estimated from linear mixed models). Notes: **, and ns represent *p* < 0.01, and not significant, respectively. Y, year; NL, N application level; GMMP, green manure mixed cropping pattern. N0, N1 and N2 indicate zero N input, 30% N reduction, and conventional N application, respectively. CK, HB, HV and RS represent sole cropped common vetch, common vetch mixed cropped with hull-less barley, common vetch mixed cropped with hairy vetch, and common vetch mixed cropped with rapeseed, respectively.

As shown in Figure 3, LMM analysis revealed that N application level, green manure mixed cropping pattern, their two-way interaction, and the three-way interaction of year × N application level × green manure mixed cropping pattern all showed highly significant effects on global warming potential (GWP) (*p* < 0.01), whereas the main effect of year had no significant influence on GWP (*p* > 0.05). Over the two experimental years, averaged across green manure patterns, N1 significantly decreased GWP by 5.27% and 14.56% compared with N0 and N2, respectively, indicating N1 as the optimal N level. Although HB, HV, and RS increased GWP by 6.27%, 10.36%, and 2.34% on average across N levels, the significant NL × GMMP interaction revealed that this effect was highly N-dependent. Specifically, under N0 and N2, all green manure treatments increased GWP (averaging ~10.4% and ~8.2%, respectively), with N0HB and N2HV showing the strongest warming effects. In sharp contrast, under N1, RS decreased GWP by 7.45%, while HB showed a negligible reduction (−0.07%). Therefore, green manure incorporation does not inevitably increase GWP; the combination of N1 rate with RS represents the optimal strategy for GWP mitigation.

**Figure 4:**
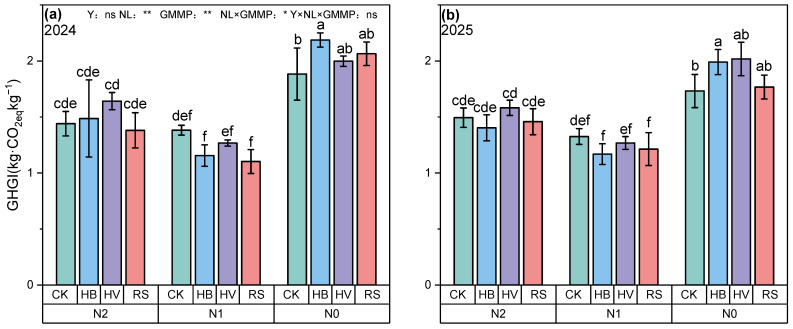
Greenhouse gas intensity (GHGI) under different treatments in 2024 (**a**) and 2025 (**b**). Different lowercase letters denote significant differences at *p* < 0.05 (Tukey’s HSD test, marginal means estimated from linear mixed models). Notes: *, **, and ns represent *p* < 0.05, *p* < 0.01, and not significant, respectively. Y, year; NL, N application level; GMMP, green manure mixed cropping pattern. N0, N1 and N2 indicate zero N input, 30% N reduction, and conventional N application, respectively. CK, HB, HV and RS represent sole cropped common vetch, common vetch mixed cropped with hull-less barley, common vetch mixed cropped with hairy vetch, and common vetch mixed cropped with rapeseed, respectively.

For greenhouse gas intensity (GHGI), as shown in Figure 4, LMM analysis showed that the main effects of N level and green manure mixed cropping pattern on GHGI were highly significant (*p* < 0.01), and their two-way interaction showed a significant effect (*p* < 0.05); whereas the main effect of year and the three-way interaction of year, N level, and green manure mixed cropping pattern had no significant effect on global warming potential (*p* > 0.05). Over the two experimental years, across all green manure mixed cropping patterns, compared with N0, the N1 treatment decreased GHGI by 36.83%; compared with N2, N1 decreased GHGI by 16.89%. Averaged across all N levels, compared with CK, the HB and HV increased GHGI by 1.43% and 5.57%, respectively, RS decreased GHGI by 2.92%. The significant NL × GMMP interaction suggested that the magnitude of green manure-induced GHGI variation varied substantially with N supply levels. Specifically, the GHGI reduction effects of green manure patterns were pronounced under N application conditions (N1 and N2) but weak under the no-N condition (N0), and the optimal green manure types for reducing GHGI differed significantly across different N regimes. Notably, the mitigation effects of optimized green manure patterns (HB and RS) on GHGI were only prominent under N reduction treatments, while green manure types had negligible effects on GHGI under zero-N conditions. Collectively, these results indicated that moderate N reduction (N1) combined with appropriate green manure patterns (e.g., HB and RS) could effectively balance field GWP and GHGI, achieving optimized greenhouse gas mitigation benefits in spring wheat production.

### 2.4. Effects of Different N Levels and Green Manure Mixed Cropping Patterns on Soil Physicochemical Properties

**Figure 5:**
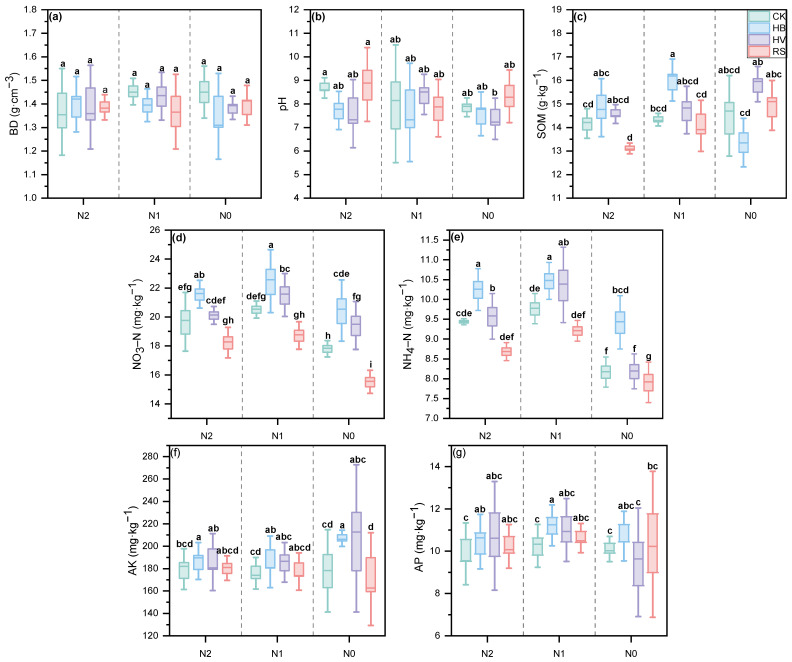
Physicochemical properties of soil under different treatment conditions in 2024. (**a**) Soil bulk density; (**b**) Soil pH; (**c**) Soil organic matter; (**d**) Nitrate nitrogen; (**e**) Ammonium nitrogen; (**f**) Available potassium; (**g**) Available phosphorus. Different lowercase letters denote significant differences at *p* < 0.05 (Tukey’s HSD test, marginal means estimated from linear mixed models). Notes: BD, bulk density; pH, soil pH; SOM, soil organic matter; NH_4_–N, ammonium nitrogen; NO_3_–N, nitrate nitrogen; AP, available phosphorus; AK, available potassium. N0, N1 and N2 indicate zero N input, 30% N reduction, and conventional N application, respectively. CK, HB, HV and RS represent sole cropped common vetch, common vetch mixed cropped with hull-less barley, common vetch mixed cropped with hairy vetch, and common vetch mixed cropped with rapeseed, respectively.

**Figure 6:**
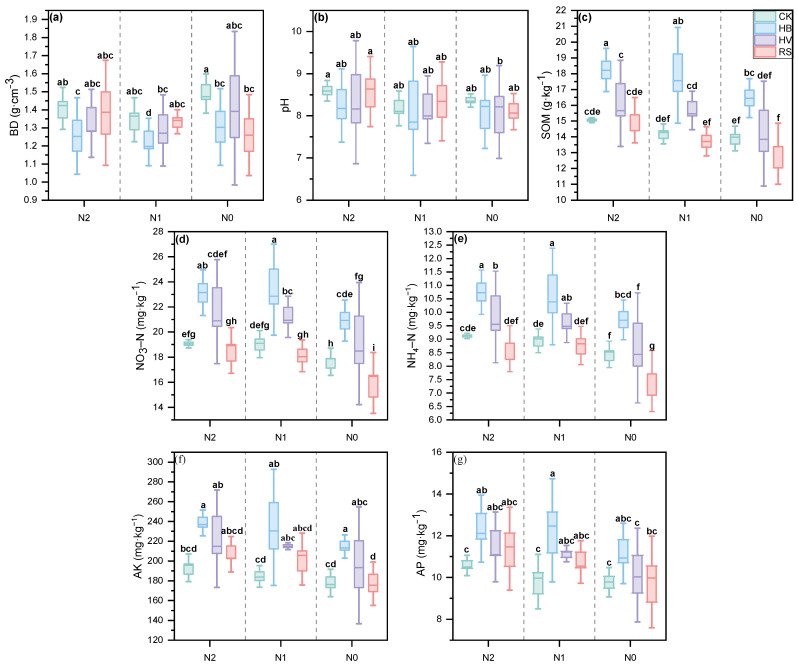
Physicochemical properties of soil under different treatment conditions in 2025. (**a**) Soil bulk density; (**b**) Soil pH; (**c**) Soil organic matter; (**d**) Nitrate nitrogen; (**e**) Ammonium nitrogen; (**f**) Available potassium; (**g**) Available phosphorus. Different lowercase letters denote significant differences at *p* < 0.05 (Tukey’s HSD test, marginal means estimated from linear mixed models). Notes: BD, bulk density; pH, soil pH; SOM, soil organic matter; NH_4_–N, ammonium nitrogen; NO_3_–N, nitrate nitrogen; AP, available phosphorus; AK, available potassium. N0, N1 and N2 indicate zero N input, 30% N reduction, and conventional N application, respectively. CK, HB, HV and RS represent sole cropped common vetch, common vetch mixed cropped with hull-less barley, common vetch mixed cropped with hairy vetch, and common vetch mixed cropped with rapeseed, respectively.

As shown in Table 2, LMM analysis showed that N application level had highly significant effects on soil organic matter (SOM), nitrate nitrogen (NO_3_–N) and ammonium nitrogen (NH_4_–N) (*p* < 0.01), and significant effects on available phosphorus (AP) (*p* < 0.05), while it had no significant effects on soil bulk density (BD), pH and available potassium (AK) (*p* > 0.05). Green manure mixed cropping pattern had highly significant effects on BD, SOM, NO_3_–N, NH_4_–N, AP and AK (*p* < 0.01), and significant effects on soil pH (*p* < 0.05). The N × pattern interaction exhibited a highly significant effect on SOM (*p* < 0.01), whereas it had no significant effects on BD, pH, NO_3_–N, NH_4_–N, AP and AK (*p* > 0.05). Year had significant effects on BD and pH (*p* < 0.05), and highly significant effects on AK and SOM (*p* < 0.01), but had no significant effects on NO_3_–N, NH_4_–N and AP (*p* > 0.05). The three-way interaction among year, N application level, and green manure mixed cropping pattern had highly significant effects on SOM and AK (*p* < 0.01), and significant effects on BD (*p* < 0.05), whereas no significant effects were observed on soil pH, NO_3_–N, NH_4_–N, or AP (*p* > 0.05). As shown in Figure 5, Figure 6 and Table A1, over the two experimental years, across all green manure mixed cropping patterns, compared with N0, the N1 treatment increased SOM, NO_3_–N, NH_4_–N, AP, and AK by 3.93%, 12.37%, 14.35%, 6.88%, and 2.11%, respectively; compared with N2, N1 increased NO_3_–N and NH_4_–N by 1.85% and 1.89%, respectively. Averaged across all N levels, compared with CK, the HB treatment increased SOM, NO_3_–N, NH_4_–N, AP, and AK by 12.40%, 15.99%, 13.59%, 12.81%, and 16.38%, respectively; the HV treatment increased SOM, NO_3_–N, NH_4_–N, AP, and AK by 5.85%, 8.13%, 4.34%, 5.86%, and 11.29%, respectively; the RS treatment increased AP and AK by 4.37% and 2.36%, but decreased SOM, NO_3_–N, and NH_4_–N by 2.65%, 7.60%, and 5.90%, respectively. These results indicate that reducing N fertilizer application combined with an appropriate green manure mixed cropping pattern can improve soil fertility.

### 2.5. Comprehensive Analysis of Soil Properties, Grain Yield, and GHGI

**Figure 7:**
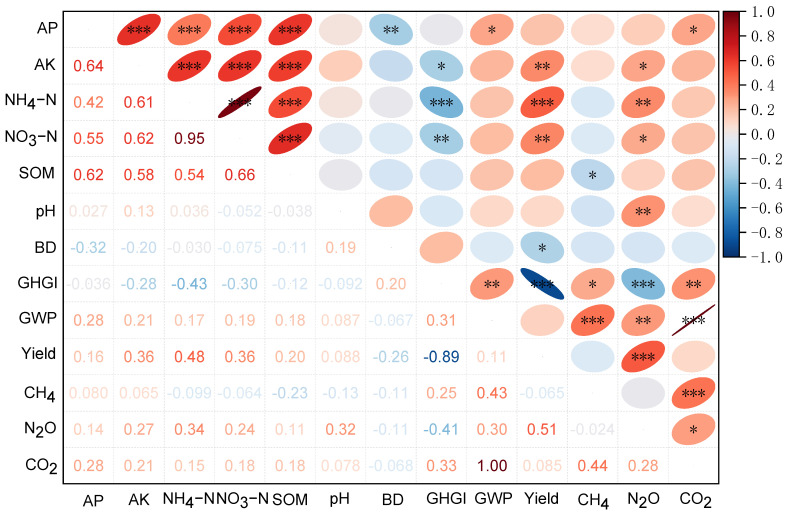
Spearman correlation heatmap of soil physicochemical properties, greenhouse gas (GHG) emissions, yield, and comprehensive indices. The color gradient represents the correlation coefficient values, with red indicating positive correlations and blue indicating negative correlations. Notes: * *p* < 0.05; ** *p* < 0.01; *** *p* < 0.001. BD, bulk density; pH, soil pH; SOM, soil organic matter; NH_4_–N, ammonium nitrogen; NO_3_–N, nitrate nitrogen; AP, available phosphorus; AK, available potassium; CO_2_, cumulative carbon dioxide emissions; N_2_O, cumulative nitrous oxide emissions; CH_4_, cumulative methane emissions; GWP, global warming potential; Yield, grain yield; GHGI, greenhouse gas intensity.

As shown in Figure 7, the Spearman correlation heatmap revealed the relationships between soil physicochemical properties, greenhouse gas fluxes, yield, and the comprehensive indices GWP and GHGI. AP and AK were significantly and positively correlated with NH_4_–N, NO_3_–N, and SOM (*p* < 0.001), indicating strong interlinkages among these key fertility indicators. NO_3_–N showed a highly strong positive correlation with NH_4_–N (*p* < 0.001), reflecting the tight link between these two mineral N pools in the soil. GHGI was significantly negatively correlated with NH_4_–N (*p* < 0.001) and NO_3_–N (*p* < 0.01), showing that the change in soil fertility has a significant impact on the GHGI values. GWP was positively correlated with cumulative CO_2_, N_2_O, and CH_4_ emissions (*p* < 0.01, *p* < 0.001, and *p* < 0.001, respectively), indicating that these three gases collectively determined GWP. Yield was positively correlated with AP, NH_4_–N, NO_3_–N, and N_2_O (*p* < 0.01 or *p* < 0.001), and negatively with BD (*p* < 0.05), implying that nutrient availability and respiration promoted productivity while compaction limited it.

**Figure 8:**
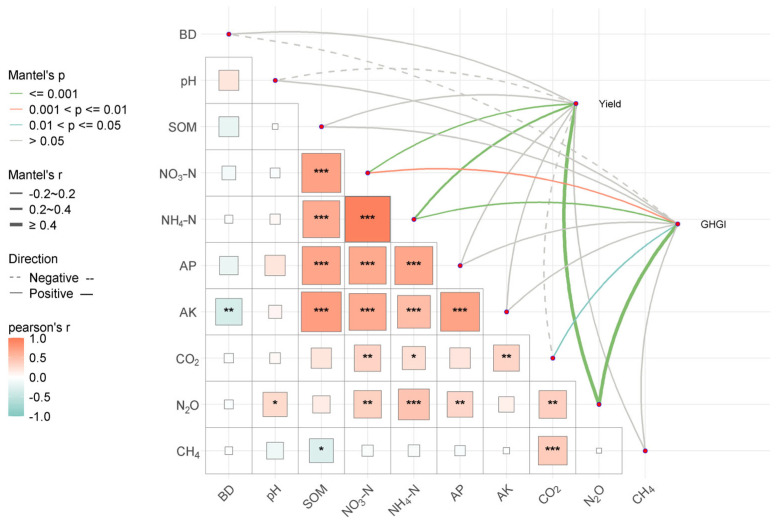
Mantel test showing correlations among soil properties, greenhouse gas (GHG) emissions, grain yield, and greenhouse gas intensity (GHGI). and line colors represent different Mantel’s p values (green: *p* ≤ 0.001; orange: 0.001 < *p* ≤ 0.01; blue: 0.01 < *p* ≤ 0.05; gray: *p* > 0.05). Square colors denote correlations among soil factors, and square sizes reflect the magnitude of these correlations (larger squares indicate stronger correlations). The analysis was performed using the R package “linkET”. Notes: * *p* < 0.05; ** *p* < 0.01; *** *p* < 0.001. BD, bulk density; pH, soil pH; SOM, soil organic matter; NH_4_–N, ammonium nitrogen; NO_3_–N, nitrate nitrogen; AP, available phosphorus; AK, available potassium; CO_2_, cumulative carbon dioxide emissions; N_2_O, cumulative nitrous oxide emissions; CH_4_, cumulative methane emissions; Yield, grain yield; GHGI, greenhouse gas intensity.

As shown in Figure 8, Mantel analysis revealed cumulative N_2_O emissions, NH_4_–N and NO_3_–N were the key factors regulating wheat yield and GHGI. Cumulative N_2_O emissions had a strong positive correlation with both wheat yield and GHGI (*r* ≥ 0.4, *p* < 0.001). NH_4_–N was moderately positively correlated with wheat yield and weakly positively correlated with GHGI (*p* < 0.001), and NO_3_–N displayed weak positive correlations with both indicators (*p* < 0.01). Cumulative CO_2_ emissions showed a weak significant positive association with GHGI (*p* < 0.05). BD, pH, SOM, AP, AK and cumulative CH_4_ emissions had no significant correlations with wheat yield or GHGI (*p* > 0.05). Mineral N fractions and cumulative N_2_O emissions served as persistent dominant predictors of yield and GHGI across the two experimental years.

**Figure 9:**
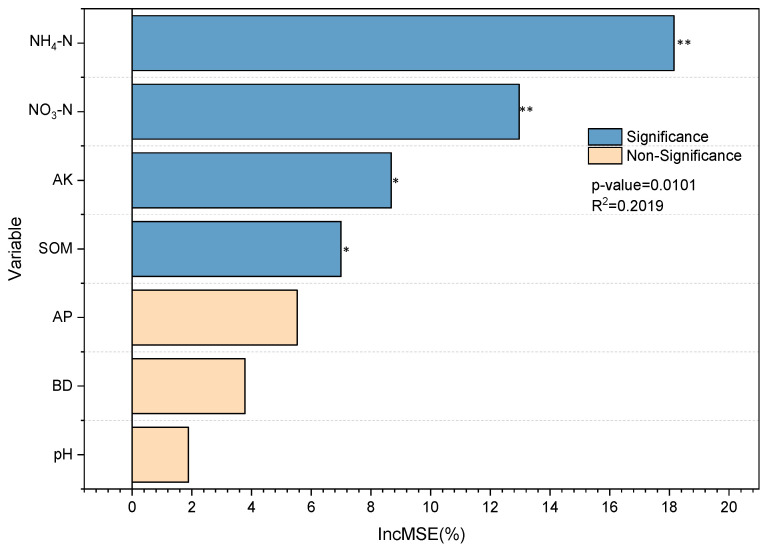
Random forest analysis identifying key factors regulating greenhouse gas intensity (GHGI). Random forest model analysis constructed using the R package “RandomForest”. The model was built with parameters ntree = 500 and default mtry, using the full dataset. Variable importance was measured as percentage increase in mean squared error. Model significance was assessed by permutation test. Notes: * *p* < 0.05; ** *p* < 0.01. BD, bulk density; pH, soil pH; SOM, soil organic matter; NH_4_–N, ammonium nitrogen; NO_3_–N, nitrate nitrogen; AP, available phosphorus; AK, available potassium.

As shown in Figure 9, random forest analysis for GHGI showed that NH_4_–N and NO_3_–N were highly significant predictors (*p* < 0.01), while AK and SOM were significant predictors (*p* < 0.05), in contrast, AP, BD, and pH were non-significant predictors (*p* > 0.05). These results indicate that NH_4_–N, NO_3_–N, AK, and SOM are key factors influencing GHGI, with NH_4_–N showing the highest importance. The importance of NH_4_–N and NO_3_–N further highlights the central role of N transformation in regulating GHGI.

## 3. Discussion

### 3.1. Mechanisms of Wheat Yield Maintenance Under Reduced N Combined with Green Manure Mixed Cropping

In this study, no significant difference in wheat grain yield was observed between the 30% N reduction treatment (N1) and the conventional N application treatment (N2), indicating that green manure mixed cropping effectively compensated for the nutrient deficit caused by N reduction. This result is consistent with conclusions from many previous studies. For example, Zhang et al. reported on the North China Plain that a 20–30% N reduction combined with green manure incorporation maintained or even increased wheat yield [42]. Similarly, Dwibedi et al. (2025) found in eastern India that replacing 25–30% N fertilizer with green manure did not affect crop yield in a rice–rapeseed rotation system [15].

The compensatory mechanisms can be attributed to several factors. First, mixed cropping of legume with gramineous or cruciferous species optimizes the overall carbon-to-nitrogen (C/N) ratio of the green manure, regulating its decomposition rate and avoiding rapid N mineralization and subsequent N loss after incorporation of legume monocultures [43]. Second, crop mixtures with different root architectures improve soil pore structure and aggregate stability, facilitating deeper root penetration and nutrient uptake [11,12]. Furthermore, green manure incorporation increases soil organic matter and microbial activity, enhancing nutrient use efficiency of subsequent crops [44]. Notably, in the present study, although the green manure pattern effect was not significant, the common vetch × hairy vetch (HV) treatment under N reduction showed a numerically higher yield than conventional N application, which may be attributed to the strong N-fixation capacity of hairy vetch and the promoting effects of its root exudates [45].

### 3.2. Regulatory Mechanisms of Greenhouse Gas Emissions Under Reduced N Combined with Green Manure Mixed Cropping

This study found that 30% N reduction (N1) compared with conventional N application (N2) significantly reduced cumulative CO_2_ emissions by 14.84%, reduced N_2_O emissions by 4.79%, and increased CH_4_ uptake by 14.13%. Compared with no N application (N0), N1 decreased CO_2_ emissions by 6.06% and increased CH_4_ uptake by 10.25%, but increased N_2_O emissions by 19.32%, indicating that N_2_O emission reduction mainly benefits from avoiding excessive N input rather than from reducing N from zero to a moderate level. These results are consistent with the global meta-analysis by Linquist et al. (2012), which concluded that N fertilizer reduction is the most effective way to mitigate N_2_O emissions from croplands [2]. Notably, under N reduction, the common vetch × rapeseed (RS) treatment further decreased CO_2_ emissions (significantly lower than CK), whereas the HV treatment increased CO_2_ emissions, highlighting the importance of selecting appropriate green manure species for climate mitigation.

However, averaged across N levels, all green manure patterns (HB, HV, RS) significantly decreased CH_4_ uptake compared with CK (by 16.74–21.89%), indicating that green manure incorporation itself may temporarily inhibit CH_4_ oxidation. This is consistent with previous findings that organic amendments can suppress soil CH_4_ oxidation by providing alternative substrates or altering methanotrophic activity [46,47]. Averaged across all N levels, all green manure patterns significantly reduced CH_4_ uptake compared with CK. Nevertheless, under the reduced N treatment (N1), the HB pattern showed no significant difference from CK, suggesting that the negative effect of green manure on CH_4_ oxidation can be partially alleviated by optimizing species combination and N input.

Although the interaction between N level and green manure pattern was not significant for N_2_O emissions, the main effect of green manure pattern on N_2_O emissions was significant, indicating that green manure species selection does influence N_2_O emissions, but this effect is consistent across N levels. This aligns with recent meta-analyses showing that green manure species differ in their effects on N_2_O emissions due to variations in residue quality and C/N ratio [48].

### 3.3. Effects on Global Warming Potential and Greenhouse Gas Intensity

In this study, 30% N reduction (N1) compared with conventional N application (N2) reduced global warming potential (GWP) by 14.56%, and compared with no N application (N0) reduced GWP by 5.27%. The greenhouse gas intensity (GHGI) was reduced by 36.83% (N1 vs. N0) and by 16.89% (N1 vs. N2). Compared with the findings of Huang et al. (2013) in the North China Plain, who reported that optimized N management reduced GWP by 51% [49], our mitigation magnitude is moderate but still substantial. The reduction in GHGI was more pronounced due to the combined effect of yield maintenance and GWP reduction.

In terms of component contributions, CO_2_ emissions constituted the dominant contributor to total GWP across all treatments. This finding aligns with prior studies, which documented that soil CO_2_ emissions accounted for 97.7% of total GWP within agricultural ecosystems [50]. Notably, under N reduction, the RS treatment showed greater mitigation effects (significantly lower GWP and CO_2_ emissions than CK), whereas the HV treatment increased GWP. The advantage of the RS treatment may stem from organic acids secreted by rapeseed roots, which regulate soil pH and affect nitrifier and denitrifier activities [51]. The HV treatment may benefit from complementary effects between two legume species, where differences in root architecture improve soil aeration [44].

Random forest analysis identified soil NH_4_–N and NO_3_–N as the core factors regulating GHGI. This result underscores that when evaluating mitigation practices, both yield effects (to avoid GHGI dilution) and soil nutrient status must be considered. Abubakar et al. (2022) identified soil NO_3_–N and NH_4_–N as the most important factors affecting GHG emissions [52]. This consistency reinforces the critical role of inorganic N availability in driving GHGI across different agroecosystems.

### 3.4. Mechanisms of Soil Physicochemical Property Improvement Under Green Manure Mixed Cropping Synergized with N Reduction

This study found that 30% N reduction (N1) significantly increased soil NH_4_–N (by 14.35% compared with N0 and by 1.89% compared with N2), NO_3_–N (by 12.37% compared with N0 and by 1.85% compared with N2), available phosphorus (by 6.88% compared with N0), and available potassium (by 2.11% compared with N0), and had no significant negative effects on BD or pH, although N level did significantly affect pH. These results are consistent with the findings of Han et al. (2021) on the Qinghai Plateau, where long-term green manure planting improved soil N supply capacity [37].

The underlying mechanisms can be attributed to several factors. First, biological N fixation by leguminous green manure replenishes the soil N pool [45]. Second, the high C/N ratio of gramineous components in mixed green manure delays N release, reducing N losses [11]. Third, organic acids and enzymes secreted by green manure roots promote the mineralization of soil organic N [12]. Fourth, green manure mixed cropping increases exogenous organic carbon input, promotes soil aggregate formation, and enhances the physical protection of organic carbon, thereby increasing soil organic matter content [18]. In the present study, the HB and HV treatments increased soil organic matter by 12.40% and 5.85% compared with CK, respectively, further supporting this mechanism. However, the RS treatment decreased SOM, NH_4_–N, and NO_3_–N compared with CK, suggesting that the choice of green manure species is critical for soil fertility improvement.

### 3.5. Limitations and Future Research Directions

Although this study demonstrates the yield-maintaining and emission-reducing effects of 30% N reduction combined with green manure mixed cropping and elucidates the underlying mechanisms, several limitations should be acknowledged. First, this study relies on only two years of data. The long-term effects of green manure mixed cropping, such as soil carbon pool accumulation and microbial community succession, need to be verified through multi-year field experiments. Second, the frequency of greenhouse gas measurements (once every 15 days) may have underestimated some short-term emission peaks, e.g., those following fertilization or rainfall events. Future studies should employ automated measurement systems (e.g., static chambers coupled with laser isotope analyzers) to improve temporal resolution. Third, this study did not directly quantify the abundance or expression activity of functional genes related to N transformation (e.g., amoA, nirK, nosZ, pmoA), limiting a deeper molecular understanding of N_2_O and CH_4_ emission regulation. Future research should integrate metagenomics or quantitative PCR to reveal the microbial ecological mechanisms through which green manure mixed cropping regulates greenhouse gas emissions. Fourth, no synchronous soil temperature and moisture data were collected during all greenhouse gas flux samplings, which restricts quantitative analysis of soil hydrothermal covariates driving CO_2_, CH_4_ and N_2_O fluxes. Although uniform irrigation, tillage and crop management across all plots guaranteed consistent soil water and thermal backgrounds among treatments, seasonal microclimate impacts on GHG dynamics could not be quantitatively decoupled. Continuous real-time soil hydrothermal monitoring will be implemented in subsequent field trials to support mechanistic interpretation of gas flux responses. Fifth, this study was conducted at only one typical ecological site on the Qinghai Plateau; the extrapolation of the conclusions should be further tested in different climatic zones (e.g., semi-arid and alpine regions) and on different soil types. Sixth, future studies should incorporate life cycle assessment (LCA) to comprehensively evaluate the carbon footprint, N footprint, and economic costs of green manure mixed cropping systems, thereby providing a more complete decision-making basis for the green and low-carbon transformation of regional agriculture. Future efforts should also explore how such LCA-derived parameters can be embedded into tiered digital MRV frameworks, combining farmer-reported data with satellite-based verification, thereby facilitating the aggregation of carbon credits and incentivizing adoption at scale.

## 4. Materials and Methods

### 4.1. Study Area Description

**Figure 10:**
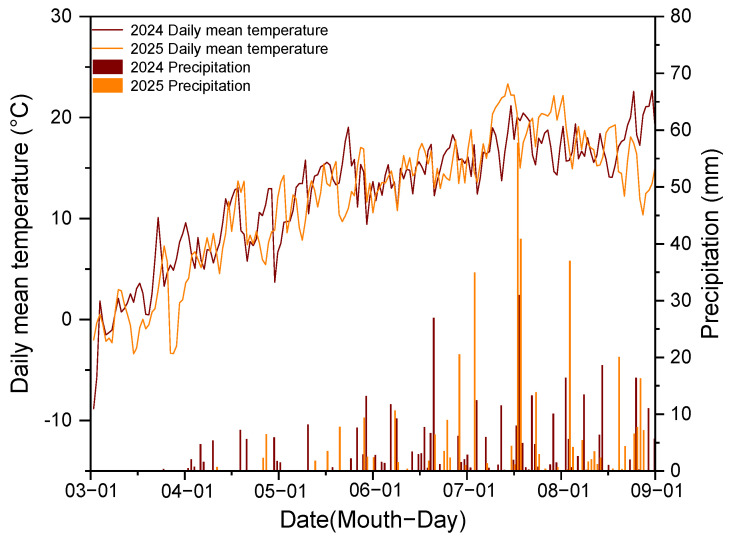
The precipitation and temperature changes during the fertility period.

The field experiment was conducted in 2024 and 2025 at the experimental base of the Academy of Agriculture and Forestry Sciences, Qinghai University, located at Mojiaquanwan, the northern suburb of Xining, Qinghai, China. The site has an average altitude of 2300 m and features a cold, continental semi-arid climate. Temperature and precipitation data are presented in Figure 10, and the annual evaporation is approximately 4.74 times the annual precipitation. Irrigation water is easily accessible from the Beichuan Canal. The soil at the experimental site is classified as Chestnut soil. In the 0–20 cm soil layer, the initial soil properties were: total N 1010.00 mg·kg^−1^, available phosphorus 12.64 mg·kg^−1^, available potassium 116.67 mg·kg^−1^, organic matter 17.37 g·kg^−1^, and pH 8.39.

### 4.2. Experimental Design

This two-year field experiment (2024–2025) was conducted to evaluate wheat responses to green manure planting and incorporation from the previous year. A split-plot design was employed. The main plots were set up with three nitrogen (N) application levels, including N0 (0 kg·ha^−1^, no N application), N1 (157.5 kg·ha^−1^, 30% reduction in the conventional N level), and N2 (225 kg·ha^−1^, conventional N level). The subplots comprised four green manure cropping patterns: common vetch sole cropping (CK), common vetch mixed with hull-less barley (HB), common vetch mixed with hairy vetch (HV), and common vetch mixed with rapeseed (RS). Spring wheat was grown in all plots before green manure planting. Each plot had an area of 15 m^2^ (5 m × 3 m). Treatments were arranged in a randomized complete block design with three replications, resulting in a total of 36 plots (3 N levels × 4 cropping patterns × 3 replicates). Guard rows (2.0 m wide) planted with spring wheat and no N fertilization were established around the experimental field to eliminate edge effects on yield measurement. Between blocks, 1.2 m-wide access paths and drainage ditches were installed to enable separate irrigation and drainage, thereby preventing surface runoff between blocks among blocks. Earthen bunds of 0.5 m width and 0.2 m height were constructed between adjacent treatments.

Fertilizer was applied only in the wheat season. Urea (46% N) was used as the N fertilizer, with 80% applied as basal fertilizer and 20% as topdressing. N topdressing was supplied when wheat plants reached the four-leaf stage. Single phosphorus fertilizer was applied at a rate of 21.6 kg P·ha^−1^ in the form of superphosphate (12% P_2_O_5_), solely as basal fertilizer. No fertilizer was applied during the green manure season.

Crop cultivars, planting density and sowing methods are listed in Table 3. Green manures were sown on 7 August 2023 and 30 July 2024, and incorporated into soil on 27 October 2023 and 29 October 2024. All aboveground green manure biomass was returned to the soil in full amount. The shoots were first fully shredded with a straw crusher, and then uniformly incorporated into topsoil using a small rotary tiller.

As shown in Table 4, The annual fresh biomass incorporated as green manure was approximately 15,000 kg·ha^−1^. As shown in Table 5, the total carbon, total N contents and C/N ratios of green manure mixtures under different treatments in 2024 and 2025 are presented.

Spring wheat was planted on 18 March 2024 and 27 March 2025, and harvested on 27 July 2024 and 30 July 2025.

Two supplementary irrigations were applied during the wheat season (100 mm each): before wheat sowing (18 March 2024 and 27 March 2025) and after N topdressing (6 May 2024 and 9 May 2025). Uniform field pest and disease management was conducted at the early tillering stage of wheat.

### 4.3. Measurement Indicators and Methods

#### 4.3.1. Collection of Greenhouse Gas Samples from Farmland

The static chamber system consists of a closed lower-opening chamber and a fixed base. The closed chamber is 100 cm in height, made of 0.5 mm thick stainless steel, with the exterior covered with thermal-insulating foam and reflective film. The bottom diameter of the chamber is 50 cm. A sampling port is installed on the top of the chamber lid, connected to a three-way stopcock for gas sampling. A small electric fan and a thermometer are mounted on the inner wall of the chamber. The fixed gas-sampling base is made of stainless steel and features a sealing water channel; it is inserted into the soil to a depth of 5 cm, with wheat plants growing inside. During measurement, the sampling chamber is placed into the base and sealed with water. While sampling, the chamber lid is closed, a portable DC power supply is connected, and the fan is activated for air mixing. Gas samples are collected using 100 mL gas bags. Sampling is conducted once every 15 days, The 15-day sampling interval was determined that each critical growth stage of spring wheat (tillering, jointing, heading, and grain filling) on the Qinghai Plateau lasts approximately 15 days. Sampling takes place between 8:30 and 11:30 a.m., with samples collected at 10, 20, and 30 min after chamber closure. The temperature inside the static chamber is recorded simultaneously. The concentrations of major greenhouse gases (CO_2_, CH_4_, N_2_O) in the gas samples are analyzed using a gas chromatograph.

#### 4.3.2. Greenhouse Gas Flux

The greenhouse gas flux was calculated using the following formula [53]:

(1) $$F=\rho \times H\times \frac{∆c}{∆t}\times \frac{273}{273+T}$$

where F is the greenhouse gas flux (CO_2_ flux in mg·m^−2^·h^−1^; CH_4_ and N_2_O fluxes in μg·m^−2^·h^−1^); ρ is the density of the gas under standard conditions (kg·m^−3^); H is the net height of the chamber (0.9 m); ∆c/∆t is the change rate of gas concentration inside the chamber (mg·m^−3^·h^−1^); and T is the average temperature inside the chamber during sampling (°C).

#### 4.3.3. Cumulative Greenhouse Gas Emissions

Cumulative greenhouse gas emissions were calculated using the following formula [54]:

(2) $$f_{n}=\sum_{i=1}^{n}F_{i}\times D_{i}$$

where $f_{n}$ is the cumulative emission of a greenhouse gas (CO_2_, CH_4_, or N_2_O) over the growing period (kg·ha^−1^); $F_{i}$ is the average daily flux between two adjacent sampling dates; and $D_{i}\;$ is the number of days between the two sampling dates.

#### 4.3.4. Global Warming Potential (GWP)

The global warming potential was calculated as follows [3]:

(3) $$GWP=E_{CO_{2}}+27\times E_{CH_{4}}+298\times E_{N_{2}O}$$

where GWP is expressed in kg CO_2_-equivalent per hectare (kg·hm^−2^), $E_{CO_{2}}$, $E_{CH_{4}}$, and $E_{N_{2}O}$ are the total emissions (or uptake, with uptake expressed as negative values) of CO_2_, CH_4_, and N_2_O over the wheat growing season, respectively. On a 100-year time horizon, the GWP conversion factors are 27 for CH_4_ and 298 for N_2_O. Negative CH_4_ fluxes (uptake) are entered as negative values in the GWP calculation.

#### 4.3.5. Greenhouse Gas Intensity (GHGI)

The greenhouse gas intensity was calculated as follows [53]:

(4) $$GHGI=\frac{GWP}{Y}$$

where GHGI is the greenhouse gas intensity (kg CO_2_-equivalent per kg grain yield), GWP is the global warming potential (kg CO_2_-equivalent·ha^−1^), and Y is the wheat grain yield (kg·ha^−1^).

#### 4.3.6. Soil Physicochemical Properties

Soil physicochemical properties were determined using the following methods:

Soil water content: oven-drying method.

Soil bulk density: cutting ring method (100 cm^3^).

Soil pH: potentiometric method.

Soil available phosphorus (AP): sodium bicarbonate extraction method.

Soil available potassium (AK): NH_4_OAc extraction-flame photometry method.

Soil organic matter (SOM) was determined by the K_2_Cr_2_O_7_ oxidation method.

Soil ammonium nitrogen and nitrate nitrogen were extracted with 2 M KCl (soil:extractant ratio = 1:10) and determined colorimetrically. NH_4_–N was measured by the indophenol blue method at 625 nm, and NO_3_–N was measured by the ultraviolet spectrophotometric method at 220 nm and 275 nm.

### 4.4. Data Processing and Analysis

Data were compiled and processed using Microsoft Office Excel 2019. Linear mixed-effects models (LMM) were fitted using the lmer function from the lme4 package in R (version 4.0.2; R Core Team, 2020) to analyze the split-plot experimental data with two nested error strata (whole-plot and subplot errors). N application level (whole-plot factor), green manure pattern (subplot factor), and their interaction were set as fixed effects, while block nested within year was specified as a random intercept (R syntax: (1|wholeid:year), where wholeid denotes the block-within-year combination). Interannual variation and treatment × year interactions were evaluated by incorporating year and all year-associated cross terms as supplementary fixed factors in a full interaction model. Data were pooled across years for subsequent analyses. Residual normality and homoscedasticity were validated via Q-Q plots, Shapiro–Wilk tests, and Levene’s tests (from the car package). For variables that did not fully satisfy normality assumptions, multiple transformations (logarithmic, square root, inverse, and Box–Cox) were tested; however, none adequately improved residual distributions. Given the robustness of LMMs to moderate violations of normality under balanced designs, and the interpretability of raw data, no transformation was applied. Pairwise comparisons of estimated marginal means with Tukey adjustment were performed using the emmeans package to identify significant differences among treatments at *p* < 0.05. Mantel tests were conducted using the linkET package. Random forest analysis was performed using the randomForest package. Graphs were created using Origin 2024 (OriginLab, Northampton, MA, USA).

## 5. Conclusions

Under a 30% N reduction, mixed green manure cropping patterns exerted divergent effects on yield, soil fertility, and greenhouse gas emissions. The common vetch × rapeseed (RS) mixture proved optimal for mitigation: it maintained wheat grain yield comparable to the conventional N level, significantly reduced cumulative CO_2_ emissions and global warming potential (GWP), and achieved the lowest greenhouse gas intensity (GHGI). In contrast, the common vetch–hull-less barley (HB) mixture excelled in yield enhancement and soil fertility improvement: it exhibited the highest grain yield among all treatments and substantially increased soil organic matter and available nutrient content, representing the optimal pattern for high productivity and soil amelioration. Random forest and Mantel analyses identified soil ammonium and nitrate nitrogen as the key edaphic drivers of GHGI, underscoring the central role of mineral N availability. Collectively, on the Qinghai–Tibet Plateau, a 30% N reduction combined with target-oriented mixed green manure patterns (RS for emission reduction, HB for high yield and fertility) offers considerable potential for low-carbon spring wheat production.

## Figures and Tables

**Table 1:** Cumulative emissions of CO_2_, CH_4_, and N_2_O under different treatments.

Year	N Application Level	Green Manure Mixed Cropping Pattern	Cumulative CO_2_ Emissions (kg·ha^−1^)	Cumulative CH_4_ Emissions (kg·ha^−1^)	Cumulative N_2_O Emissions (kg·ha^−1^)
2024	N2	CK	7867.92 ± 146.69 cde	−1.57 ± 0.01 fg	1.15 ± 0.01 ab
		HB	8570.52 ± 541.57 b	−0.67 ± 0.06 a	1.14 ± 0.06 ab
		HV	9068.48 ± 166.51 a	−1.23 ± 0.03 cde	1.13 ± 0.03 ab
		RS	8621.34 ± 356.92 b	−1.05 ± 0.01 bcd	1.17 ± 0.01 a
	N1	CK	7199.57 ± 78.05 e	−1.54 ± 0.01 fg	1.11 ± 0.01 ab
		HB	7260.21 ± 139.76 e	−1.51 ± 0.02 fg	1.06 ± 0.01 b
		HV	7914.2 ± 212.77 bcd	−1.40 ± 0.03 efg	1.07 ± 0.03 ab
		RS	6468.14 ± 221.15 f	−1.25 ± 0.03 bcde	1.08 ± 0.03 ab
	N0	CK	7195.06 ± 292.40 def	−1.57 ± 0.02 g	0.94 ± 0.02 c
		HB	8215.47 ± 193.45 bc	−1.01 ± 0.01 bc	0.88 ± 0.01 d
		HV	7530.74 ± 132.16 de	−1.25 ± 0.04 de	0.91 ± 0.04 cd
		RS	8285.08 ± 66.44 abc	−1.34 ± 0.01 ef	0.89 ± 0.04 cd
2025	N2	CK	8181.90 ± 238.68 b	−1.28 ± 0.10 de	1.15 ± 0.01 ab
		HB	8540.68 ± 338.68 ab	−1.49 ± 0.10 cde	1.13 ± 0.09 ab
		HV	8957.29 ± 200.35 a	−1.24 ± 0.17 ab	1.17 ± 0.05 ab
		RS	8426.15 ± 192.88 ab	−1.23 ± 0.06 ab	1.14 ± 0.02 a
	N1	CK	7293.61 ± 218.49 cde	−1.53 ± 0.09 de	1.12 ± 0.02 ab
		HB	7248.97 ± 346.18 cde	−1.51 ± 0.10 de	1.07 ± 0.03 b
		HV	7822.6 ± 319.49 bcd	−1.41 ± 0.06 bcde	1.12 ± 0.05 ab
		RS	6899.18 ± 353.97 e	−1.29 ± 0.12 abc	1.10 ± 0.05 ab
	N0	CK	7111.37 ± 178.65 de	−1.56 ± 0.06 e	0.95 ± 0.03 c
		HB	7953.71 ± 298.11 bc	−1.30 ± 0.07 bcd	0.89 ± 0.01 d
		HV	8349.58 b ± 569.47 ab	−1.24 ± 0.06 abc	0.96 ± 0.07 cd
		RS	7216.74 ± 139.79 de	−1.11 ± 0.08 a	0.92 ± 0.06 cd
Year			ns	*	ns
N application level			**	**	**
Green manure mixed cropping pattern			**	**	**
N application level × Green manure mixed cropping pattern			**	**	ns
Year × N application level × Green manure mixed cropping pattern			**	**	ns

Different lowercase letters denote significant differences at *p* < 0.05 (Tukey’s HSD test, marginal means estimated from linear mixed models). Notes: *, **, and ns represent *p* < 0.05, *p* < 0.01, and not significant, respectively. N0, N1 and N2 indicate zero N input, 30% N reduction, and conventional N application, respectively. CK, HB, HV and RS represent sole cropped common vetch, common vetch mixed cropped with hull-less barley, common vetch mixed cropped with hairy vetch, and common vetch mixed cropped with rapeseed, respectively. Negative values indicate net CH_4_ uptake by the soil. All comparisons of CH_4_ are based on the magnitude of uptake (i.e., the absolute value).

**Table 2:** Interaction analysis of N application levels and green manure mixed cropping patterns on soil physicochemical properties.

Indicators	Year	N Application Level	Green Manure Mixed Cropping Pattern	N Application Level × Green Manure Mixed Cropping Pattern	Year × N Application Level × Green Manure Mixed Cropping Pattern
BD	*	ns	**	ns	*
pH	*	ns	*	ns	ns
SOM	**	**	**	**	**
NO_3_–N	ns	**	**	ns	ns
NH_4_–N	ns	**	**	ns	ns
AP	ns	*	**	ns	ns
AK	**	ns	**	ns	**

Notes: *, **, and ns represent *p* < 0.05, *p* < 0.01, and not significant, respectively. BD, bulk density; pH, soil pH; SOM, soil organic matter; NO_3_–N, nitrate nitrogen; NH_4_–N, ammonium nitrogen; AP, available phosphorus; AK, available potassium.

**Table 3:** Crop cultivars, planting density and sowing methods.

Crop	Variety	Planting Density (kg·ha^−1^)	Seeding Method
spring wheat	Qingchun 38	300	Drilling (row spacing 15 cm)
common vetch	Ximu 333	225/180	Broadcasting
hull-less barley	Kunlun 15	52.5	Broadcasting
hairy vetch	Turkmen	30	Broadcasting
rapeseed	Qingza 9	1.5	Broadcasting

Note: The seeding rate of common vetch was 225 kg·ha^−1^ in the sole cropped common vetch treatment. For mixed cropping treatments of vetch with hull-less barley, hairy vetch and rapeseed, the seeding rate of vetch was adjusted to 180 kg·ha^−1^, while the corresponding seeding rates of hull-less barley, hairy vetch and rapeseed were 52.5, 30 and 1.5 kg·ha^−1^, respectively.

**Table 4:** Dry and fresh weights of green manure mixtures under different treatments in 2024 and 2025.

N Application Level	Green Manure Mixed Cropping Pattern	2024		2025	
		Fresh Weight of Green Manure (kg·ha^−1^)	Dry Weight of Green Manure (kg·ha^−1^)	Fresh Weight of Green Manure (kg·ha^−1^)	Dry Weight of Green Manure (kg·ha^−1^)
N2	CK	15,218	6284	15,819.11	6400.933
	HB	16,806	6972	17,293.37	7030.001
	HV	16,470	6924	16,794.46	6918.503
	RS	16,138	6780	16,436.55	6766.645
N1	CK	15,425	6301	15,728.87	6295.997
	HB	16,658	6651	16,491.42	6452.155
	HV	16,205	6391	16,334.64	6312.654
	RS	15,977	5988	15,937.06	5852.984
N0	CK	13,714	5237	14,070.56	5265.172
	HB	15,166	5575	15,446.57	5564.018
	HV	15,571	5817	15,075.84	5518.827
	RS	13,852	5450	14,253.71	5495.339

**Table 5:** Total carbon, total N, and C/N ratios of green manure mixtures under different treatments in 2024 and 2025.

Green Manure Mixed Cropping Pattern	Content	2024	2025
CK	TC (%)	45.20	45.13
	TN (%)	3.92	3.87
	C/N ratio	11.53	11.66
HB	TC (%)	52.50	51.96
	TN (%)	3.33	3.36
	C/N ratio	15.76	15.46
HV	TC (%)	44.54	45.31
	TN (%)	3.78	3.75
	C/N ratio	11.78	12.08
RS	TC (%)	46.50	46.84
	TN (%)	3.57	3.53
	C/N ratio	13.02	13.26

## Data Availability

The original contributions presented in this study are included in the article. Further inquiries can be directed to the corresponding author.

## Appendix A

**Table A1:** Physicochemical properties of soil under different treatment conditions in 2024 and 2025.

Year	N Application Level	Green Manure Mixed Cropping Pattern	BD	pH	SOM	NO_3_–N	NH_4_–N	AK	AP
2024	N2	CK	1.37 ± 0.07 a	8.68 ± 0.17 a	14.17 ± 0.25 cd	19.67 ± 0.81 efg	9.44 ± 0.03 cde	179.60 ± 7.33 bcd	9.87 ± 0.59 c
		HB	1.40 ± 0.05 a	7.72 ± 0.33 ab	14.84 ± 0.50 abc	21.57 ± 0.39 ab	10.25 ± 0.21 a	186.84 ± 6.64 a	10.45 ± 0.52 ab
		HV	1.39 ± 0.07 a	7.59 ± 0.58 ab	14.57 ± 0.16 abcd	20.11 ± 0.25 cdef	9.57 ± 0.23 b	185.86 ± 10.21 ab	10.73 ± 1.03 abc
		RS	1.39 ± 0.02 a	8.83 ± 0.63 a	13.11 ± 0.09 d	18.23 ± 0.42 gh	8.69 ± 0.09 def	180.47 ± 4.44 abcd	10.22 ± 0.41 abc
	N1	CK	1.45 ± 0.02 a	8.01 ± 1.01 ab	14.33 ± 0.11 bcd	20.51 ± 0.24 defg	9.77 ± 0.15 de	175.81 ± 5.67 cd	10.26 ± 0.41 c
		HB	1.39 ± 0.03 a	7.64 ± 0.84 ab	16.02 ± 0.36 a	22.47 ± 0.88 a	10.47 ± 0.19 a	186.03 ± 9.27 ab	11.22 ± 0.39 a
		HV	1.43 ± 0.04 a	8.41 ± 0.34 ab	14.74 ± 0.40 abcd	21.52 ± 0.60 bc	10.37 ± 0.38 ab	185.61 ± 7.12 abc	11.00 ± 0.60 abc
		RS	1.37 ± 0.06 a	7.82 ± 0.49 ab	14.07 ± 0.44 cd	18.72 ± 0.38 gh	9.21 ± 0.10 def	177.37 ± 6.71 abcd	10.62 ± 0.28 abc
	N0	CK	1.45 ± 0.04 a	7.86 ± 0.16 ab	14.50 ± 0.69 abcd	17.81 ± 0.23 h	8.17 ± 0.15 f	178.01 ± 14.77 cd	10.10 ± 0.24 c
		HB	1.35 ± 0.07 a	7.58 ± 0.37 ab	13.36 ± 0.42 cd	20.45 ± 0.85 cde	9.42 ± 0.27 bcd	207.05 ± 2.92 a	10.72 ± 0.47 abc
		HV	1.38 ± 0.02 a	7.37 ± 0.35 b	15.84 ± 0.30 ab	19.42 ± 0.67 fg	8.19 ± 0.18 f	206.99 ± 26.48 abc	9.48 ± 1.03 c
		RS	1.39 ± 0.03 a	8.32 ± 0.45 ab	14.94 ± 0.43 abc	15.53 ± 0.32 i	7.91 ± 0.21 g	170.65 ± 16.64 d	10.33 ± 1.39 bc
2025	N2	CK	1.41 ± 0.05 ab	8.60 ± 0.10 a	15.06 ± 0.06 cde	19.07 ± 0.13 efg	9.12 ± 0.04 cde	193.19 ± 5.61 bcd	10.58 ± 0.20 c
		HB	1.26 ± 0.09 c	8.24 ± 0.35 ab	18.23 ± 0.55 a	23.13 ± 0.73 ab	10.75 ± 0.33 a	238.49 ± 5.24 a	12.34 ± 0.65 ab
		HV	1.33 ± 0.08 abc	8.32 ± 0.59 ab	16.12 ± 1.10 c	21.63 ± 1.67 cdef	9.83 ± 0.68 b	222.55 ± 19.80 ab	11.46 ± 0.67 abc
		RS	1.38 ± 0.12 abc	8.57 ± 0.33 a	15.07 ± 0.58 cde	18.54 ± 0.73 gh	8.65 ± 0.34 def	206.91 ± 7.21 abcd	11.38 ± 0.80 abc
	N1	CK	1.35 ± 0.05 abc	8.18 ± 0.17 ab	14.19 ± 0.25 def	19.04 ± 0.43 defg	8.94 ± 0.18 de	184.42 ± 4.42 cd	9.81 ± 0.53 c
		HB	1.22 ± 0.05 d	8.12 ± 0.62 ab	17.90 ± 1.22 ab	23.37 ± 1.46 a	10.59 ± 0.72 a	234.03 ± 23.61 ab	12.26 ± 1.00 a
		HV	1.29 ± 0.08 bc	8.15 ± 0.32 ab	15.67 ± 0.49 cd	21.21 ± 0.66 bc	9.60 ± 0.29 ab	215.00 ± 1.42 abc	11.15 ± 0.16 abc
		RS	1.33 ± 0.03 abc	8.34 ± 0.38 ab	13.72 ± 0.37 ef	18.11 ± 0.51 gh	8.77 ± 0.28 def	202.00 ± 10.56 abcd	10.74 ± 0.41 abc
	N0	CK	1.49 ± 0.04 a	8.36 ± 0.06 ab	13.90 ± 0.31 ef	17.63 ± 0.44 h	8.44 ± 0.20 f	177.77 ± 5.57 cd	9.78 ± 0.28 c
		HB	1.31 ± 0.09 bc	8.10 ± 0.35 ab	16.45 ± 0.49 bc	20.91 ± 0.66 cde	9.72 ± 0.30 bcd	214.69 ± 4.72 a	11.15 ± 0.58 abc
		HV	1.41 ± 0.17 abc	8.09 ± 0.44 b	14.20 ± 1.33 def	19.09 ± 1.96 fg	8.68 ± 0.82 f	195.72 ± 23.79 abc	10.12 ± 0.91 c
		RS	1.26 ± 0.09 bc	8.10 ± 0.17 ab	12.94 ± 0.78 f	15.96 ± 0.98 i	7.45 ± 0.46 a	177.11 ± 8.82 d	9.79 ± 0.88 bc

Different lowercase letters denote significant differences at *p* < 0.05 (Tukey’s HSD test, marginal means estimated from linear mixed models). Notes: N0, N1 and N2 indicate zero N input, 30% N reduction, and conventional N application, respectively. CK, HB, HV and RS represent sole-cropped common vetch, common vetch mixed cropped with hull-less barley, common vetch mixed cropped with hairy vetch, and common vetch mixed cropped with rapeseed, respectively.
